# Synthesis and Characterization of Surfactant for Retarding Acid–Rock Reaction Rate in Acid Fracturing

**DOI:** 10.3389/fchem.2021.715009

**Published:** 2021-08-20

**Authors:** Fuli Yan, Yongmin Shi, Yu Tian

**Affiliations:** ^1^ Shaanxi Key Laboratory of Chemical Additives for Industry, College of Chemistry and Chemical Engineering, Shaanxi University of Science and Technology, Xi’an, China; ^2^ School of Earth and Space Sciences, Peking University, Beijing, China

**Keywords:** surfactant, viscosity, retarding reaction rate, retarding reaction mechanism, acid fracturing

## Abstract

Acid fracturing is an effective method to develop ultra-low permeability reservoirs. However, the fast reaction rate reduces the effect of the acid fracturing and increases the near-well collapse risk. Therefore, it is necessary to retard the acid–rock reaction rate. In this work, we synthesized an acid-resistant Gemini zwitterionic viscoelastic surfactant (named VES-c), which has good performances such as temperature resistance, salt resistance, and shear resistance. Besides, a low concentration of VES-c increases the viscosity of the acid solution. The CO_2_ drainage method was used to measure the reaction rate between the dibasic acid and dolomite/broken core. We find that the dibasic acid containing 0.3% VES-c retards the dolomite reaction rate of 3.22 times compared with only dibasic acid. Furthermore, the dibasic acid containing 0.3% VES-c exhibits uniform distribution and is not easy to adhere to the solid surface. The VES-c also is favorable to reduce the formation of amorphous calcium carbonate. Retarding the rate of acid–rock reaction and enhancing the acidification are mainly attributed to VES-c's salt-tolerance, anti-adsorption, and the property of increasing the viscosity of the solution. Hopefully, this kind of surfactant retarding reaction rate is applied to other acid–rock reactions.

## Introduction

Conventional oil fields have entered the middle and late stages of exploitation after years of development, but there are still low and difficult exploitation potentials. The proportion of reserves in ultra-low permeability oil fields has been increasing year by year. Therefore, the development of ultra-low permeability reservoirs becomes important (Guo et al., 2017), and the main means of development is fracturing techniques. Since 1947, the hydraulic fracturing fluid technique was first used in the Kansas–Houghton field (PAK. and Chan, 2004), and the fracturing fluid technique has received considerable attention. Subsequently, other fracturing fluid techniques were greatly developed, for example, hydraulic fracturing fluids (Zhang et al., 2018a; Zhou et al., 2019), oil-based fracturing fluid (Zhang et al., 2018b), emulsified fracturing fluid (Buijse and van Domelen, 2000; Sayed et al., 2012; Zakaria and Nasr-EI-Din, 2015), foam fracturing fluid (Sayed and Al-Muntasheri, 2016; Dehdari et al., 2020; Qu et al., 2020), thickening fracturing fluid (Liu and Li, 2016; Cai et al., 2018), alcohol-based hydraulic fracturing (Marrugo-Hernandez et al., 2018), and surfactant fracturing fluid (Zhang et al., 2018c; Lu et al., 2019; Mejia et al., 2019; Tangirala and Sheng, 2019; Zhang et al., 2019). Although hydraulic fracturing fluid has been widely used, it still represents the poor stability to shear resistance and serious filtration loss. To this end, surfactant fracturing fluid has been developed in recent decades. This kind of surfactant fracturing fluid shows good performance, such as shear resistance, temperature resistance, salt resistance, and harmlessness to reservoirs (Yu et al., 2019a; Chen et al., 2019). Acid fracturing is a widely used technique in both new and existing wells to increase the production in ultra-low permeability reservoirs (Rbeawi et al., 2018).

Usually, the minerals composition of the reservoir mainly includes illite, chlorite, montmorillonite, kaolinite, calcite, laumontite, dolomite, quartz, feldspar, and muscovite. The corresponding chemical composition of each mineral is summarized in Supplementary Table S1. Most minerals react with acid, especially the carbonate minerals (e.g., calcite and dolomite), leading to dissolving fillings in the reservoirs and reducing the compressive strength of reservoir rocks (Zhang and Fang, 2020).

In heterogeneous tight reservoirs, a large permeability contrast results in fluids flowing into the highly permeable zone, which does not effectively cover the tight target reservoir, thus reducing the overall efficiency of acid fracturing measures. To improve the cleaning efficiency of acid on reservoir interstitial materials, the polymer and viscoelastic surfactant (Afra et al., 2020) are used to increase the viscosity of the acid solution, reduce fluid loss, and prolong the distance of acid etching (Jones and Dovle, 1996). Polymers have good temperature resistance and shear resistance, but polymer solutions need strong oxidants (e.g., ammonium persulfate and potassium persulfate) as gel-breaking (Wang et al., 2016). Gel-breaking oxidants oxidize Fe^2+^ and produce its precipitation, causing secondary damage to the reservoir. Besides, the polymers do not break gel easily and adhere to the surface of the rock, causing damage to the reservoir. Therefore, surfactants have attracted the researchers’ attention to provide a clean fracturing fluid because of their small molecular weights and there is no need for a gel breaker.

In this work, an acid-resistant Gemini zwitterionic viscoelastic surfactant (VES-c) was synthesized. A low concentration of VES-c can effectively retard the acid–rock reaction rate and increase the effect of rock acidification. Moreover, the VES-c does not adhere easily to the rock surface.

## Results and Discussion

### VES-c Synthesis

**SCHEME 1:**
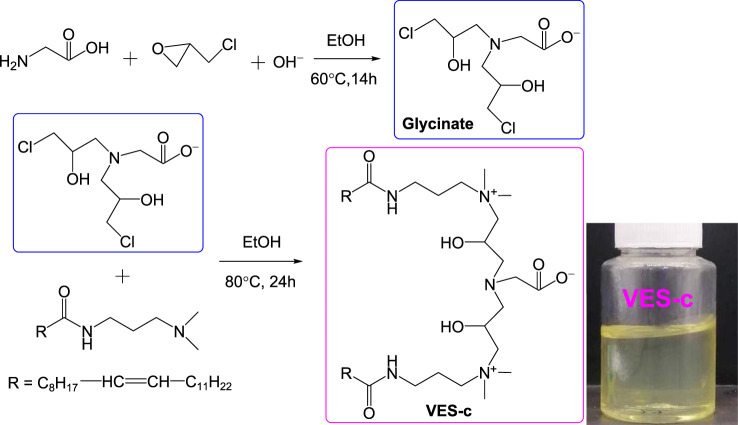
Synthesis route of VES-c surfactant and the VES-c sample.

Scheme 1 shows the synthesis route of the VES-c. First, the intermediate (glycinate) was synthesized by the reaction of epichlorohydrin and glycine. A 3.75 g (50.0 mmol) of glycine was dissolved with 100 ml deionized water in a 500 ml flask. Epichlorohydrin of 7.87 ml (100.48 mmol) dissolved in 30 ml ethanol was quickly poured into the glycine solution. Sodium hydroxide (50.0 mmol) was added to the mixed solution. The flask was moved to an oil bath and heated at 60°C with stirring for 14 h. Second, erucamidopropyl dimethylamine of 42.24 g (100.1 mmol) was dissolved in 40 ml ethanol in a beaker, and the ethanol solution was poured into the flask containing the intermediate solution. Besides, 30 ml ethanol was used to wash the beaker three times, and the washing liquids were also poured into the flask. The flask was moved to the oil bath and heated at 80°C with stirring for 24 h. Finally, the negative pressure rotary evaporation was conducted to remove the solvent (water and ethanol) and obtain the crude product. Then, recrystallization was conducted three times to purify the product using ethanol and acetone mixture (volume ratio: 1/3). The other materials and methods are collected in the Supplementary Material.

### Measuring Rate of Acid–Rock Reaction

In acid fracturing, one or more acids such as hydrofluoric acid, hydrochloric acid, fluoroboric acid, acetic acid, and formic acid are usually used (Yang et al., 2006; Assem et al., 2019; Jeffry et al., 2020). In addition, the F^−^ in the hydrofluoric acid and fluoroboric acid reacts with Ca^2+^, Mg^2+^, and Fe^3+^ to form water-insoluble salts, which are not selected. Formic acid is toxic and not easy to operate. Hydrochloric acid and acetic acid can react with carbonate minerals, and Cl^−^ and CH_3_COO^−^ do not form insoluble salts with cations. Therefore, we selected hydrochloric acid and acetic acid. Due to the fast reaction of the hydrochloric acid with rock minerals, producing fragments or broken particles of rock minerals and damage in the reservoir could occur during the acidification process. Acetic acid is a good choice for acid fracturing because acetic acid has a slower reaction with rock minerals than hydrochloric acid. To improve the efficiency of acid fracturing, we used dibasic acid, including hydrochloric acid and acetic acid.

**SCHEME 2:**
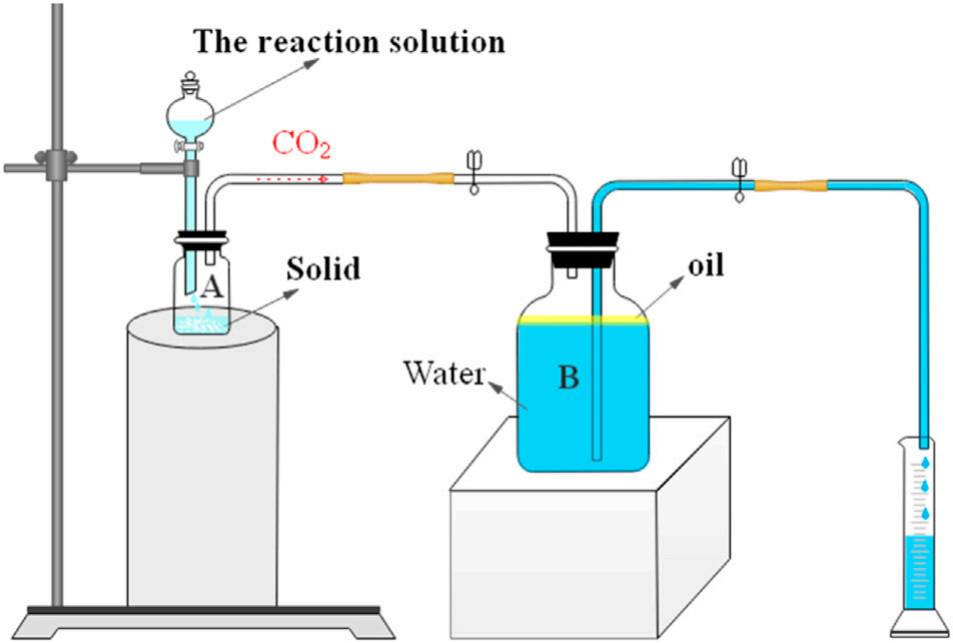
Measuring the reaction rate using the volume of drained water by the acid–rock reaction.

Acidifying tests usually use acid-resistant core displacement devices (Wang et al., 2020) and acid-resistant simulated fracturing devices (Asadollahpour et al., 2019). The cost of those devices is huge and inconvenient in the laboratory. In this study, the reaction rate was measured through the self-assembled device (see Scheme 2). The reaction rate of 0.3% VES-c dibasic acid and rock was calculated by measuring the amount of CO_2_ produced. The CO_2_ enters the sealed container B, where the oil is added to prevent CO_2_ from dissolving in water. As the reaction proceeds, the pressure in the B bottle increases, and the water is drained into the cylinder. The reaction rate is calculated by the volume of drained water divided by the reaction time.

Compared with the core displacement device or the simulated acidizing fracturing device, the disadvantage of our self-assembled device is that it is insufficient to achieve the experimental process pressurization operation and the simulated fracturing operation. However, the advantages are that the device is cheap, simple, convenient, and easy to operate and can be assembled at any time in the laboratory. In addition, the reaction of the acid and solid can be observed intuitively, which is more suitable to study whether the synthesized VES-c can delay the reaction rate.

### Structural Characterization

#### FT-IR

**FIGURE 1:**
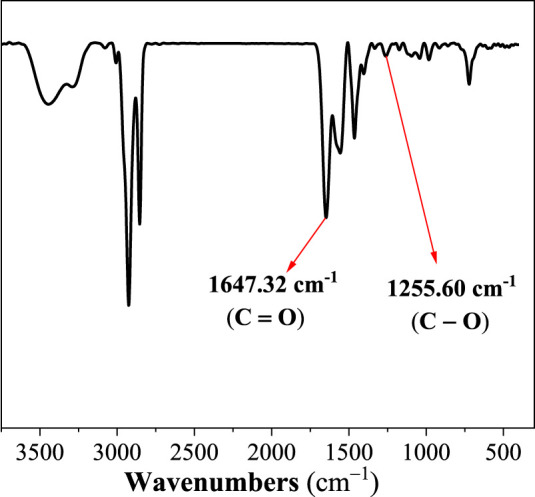
The infrared spectrum of VES-c.

To determine the structure of VES-c, the FT-IR spectrum is performed, and the result is shown in Figure 1. We observe the stretch vibration absorption peak of C=O at 1647.32 cm^−1^ and the stretch vibration peak of C–O at 1255.60 cm^−1^. This indicates that the carboxylate is successfully connected. Besides, the peak at 3278.84 cm^−1^ is the stretch vibration absorption peak of O–H. The peaks at 3440.50 and 1548.61 cm^−1^ represent the stretch and bending vibration absorption peaks of amide N–H. The peaks at 3005.38, 2927.44, and 2854.72 cm^−1^ are the stretch vibration absorption peaks of C–H, –CH_3_, and –CH_2_–, respectively. The peaks at 964.37, 727.13, and 576.69 cm^−1^ are the bending vibration absorption of C–H, –CH_3_, and –CH_2_–, respectively.

#### NMR

**FIGURE 2:**
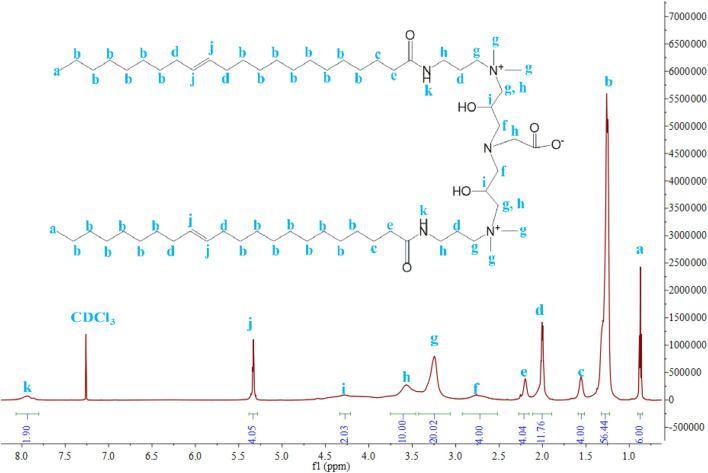
^1^H NMR of VES-c.

^1^H NMR and ^13^C NMR were used to confirm the structure of VES-c and verify the purity of VES-c. Figure 2 shows the ^1^H NMR (600 MHz, chloroform-d) spectrum of VES-c:7.93 (s, 2H), 5.33 (t, J = 5.0 Hz, 4H), 4.28 (s, 2H), 3.75–3.46 (m, 10H), 3.25 (s, 20H), 2.76 (s, 4H), 2.20 (s, 4H), 2.00 (q, J = 6.6 Hz, 12H), 1.56 (s, 4H), 1.27 (td, J = 15.1, 13.0, 7.7 Hz, 56H), and 0.87 (t, J = 6.9 Hz, 6H). Supplementary Figure S1 shows the ^13^C NMR (151 MHz, Chloroform-d) spectrum of VES-c: δ 174.95, 129.99, 129.90, 129.89, 52.44, 52.27, 51.87, 36.55, 32.05, 30.09, 30.04, 29.98, 29.95, 29.92, 29.82, 29.77, 29.69, 29.65, 29.62, 29.47, 27.42, 27.36, 26.10, 22.82, and 14.25.

There are almost no impurity peaks in Figure 2 and Supplementary Figure S1, indicating that the VES-c sample is relatively pure.

### VES-c Surface Tension

**FIGURE 3:**
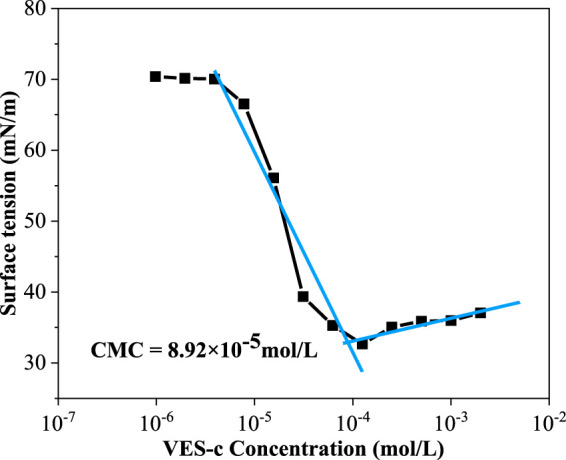
Surface tension plot for the VES-c solution.

The surface tension (γ) values of various concentrations of VES-c solution were measured at 25°C. In the low concentration range (9.7 × 10^−7^ – 3.9 × 10^−6^ mol/L), the γ value is close to the γ value of deionized water (γ_deionized water_ = 72.286 mN/m), and with the increase of VES-c concentration, the γ value greatly decreases and finally approaches to a certain value, as shown in Figure 3. The critical point is obtained by the intersection of two linear fittings. The concentration of VES-c at this point is known as the critical micelle concentration (CMC). At the CMC point, the surfactant molecules in the solution begin to form micelles. The CMC of VES-c was 89.2 μmol/L, and the corresponding *γ*_CMC_ was 32.8 mN/m, indicating that VES-c increases the viscosity of the solution at a low concentration.

### VES-c Dissolution

The dissolution of 0.3% VES-c, 1.5% VES-c, and 2.7% VES-c in different concentrations of hydrochloric acid solutions and different concentrations of NaCl solutions was analyzed, respectively. The results show that different concentrations of VES-c are well dissolved in deionized water, hydrochloric acid solutions, and NaCl solutions. Besides, the viscosity of VES-c acid solutions or VES-c NaCl solutions is higher than that of deionized water by conducting a vial inversion test. The detailed figures are collected in Supplementary Figure S2.

### VES-c Viscosity

**FIGURE 4:**
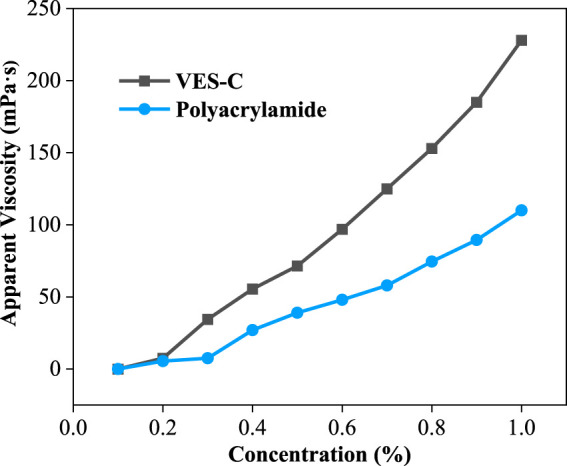
The comparison of viscosities between the VES-c and polyacrylamide.

The viscosity of VES-c and polyacrylamide were compared at the same concentration (see Figure 4). After the concentration of 0.2%, VES-c solutions’ viscosity became higher than that of polyacrylamide solution. More importantly, the VES-c (relative molecular mass of 1032) shows good performance to the reservoir and environment compared to polyacrylamide (8–10 million relative molecular mass).

### VES-c Shear Resistance

**FIGURE 5:**
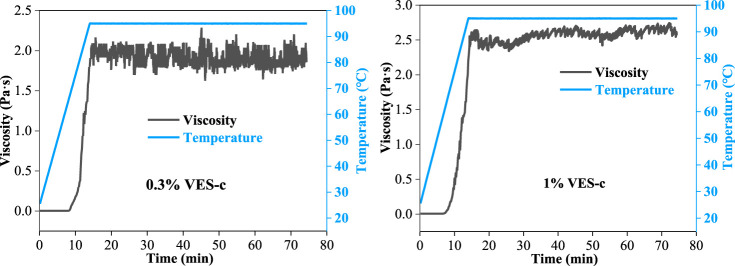
Variation of the VES-c solution’s viscosity under 170 s^−1^ continuous shear rate at temperatures from 25°C to 95°C.

The injected liquid is affected by the frictional shear of the pipe wall and the reservoir rock; thus, it requires that the solution has a good shear resistance. The shear resistance of 0.3% VES-c and 1% VES-c solutions was measured by the dynamic rheometer at the temperature from 25°C to 95°C and the shear rate of 170 s^−1^ (see Figure 5). Before 60°C, the shear viscosities of 0.3% VES-c and 1% VES-c solutions are both small (close to 0 Pa·s). When the temperature exceeds 60°C, their shear viscosities greatly increase with temperatures rising because increasing the temperature facilitates the entanglement motion of VES-c molecules. After 75 min, the viscosity values still have a small fluctuation range, which shows that the VES-c has good shear resistance.

### VES-c Temperature Resistance

**FIGURE 6:**
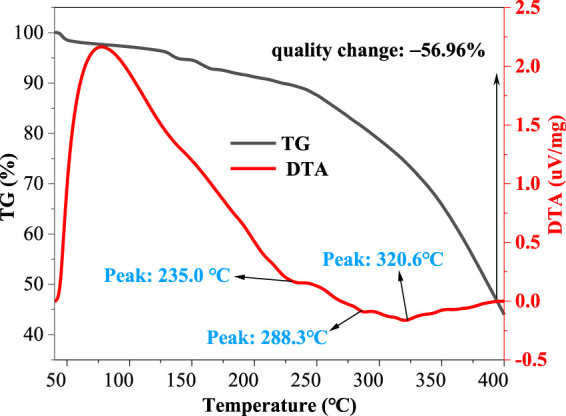
Thermal analysis diagram of VES-c.

To explore the temperature resistance of VES-c, we used the synchronous thermal analyzer to measure the VES-c solution in the temperature ranges from 40°C to 400°C. Figure 6 shows that, at 235.0°C, the first peak of the DTA curve appears, indicating that the first endothermic decomposition of VES-c occurs. When the temperature reached 400°C, the mass of VES-c was reduced by 56.96%. It means that the VES-c has good temperature resistance and can apply to high-temperature reservoirs. However, whether the components after chain scission continue to exert the effect of surfactant needs future experimental verification, which is beyond this work.

### VES-c Microstructure

In the previous reports, there are three main forms of surfactants in dilute solutions: spherical micelle, rod-shaped micelles, and spherical bilayer vesicles (Israelachvili and Mitchell, 1976). As the concentration increases, a large number of surfactant molecules aggregate to form densely structured worm-like micelles (see Supplementary Figure S3). The worm-like micelles are entangled with each other and increase the viscoelasticity of the solution (Bulgakova et al., 2013; Yang and Hou, 2020).

**FIGURE 7:**
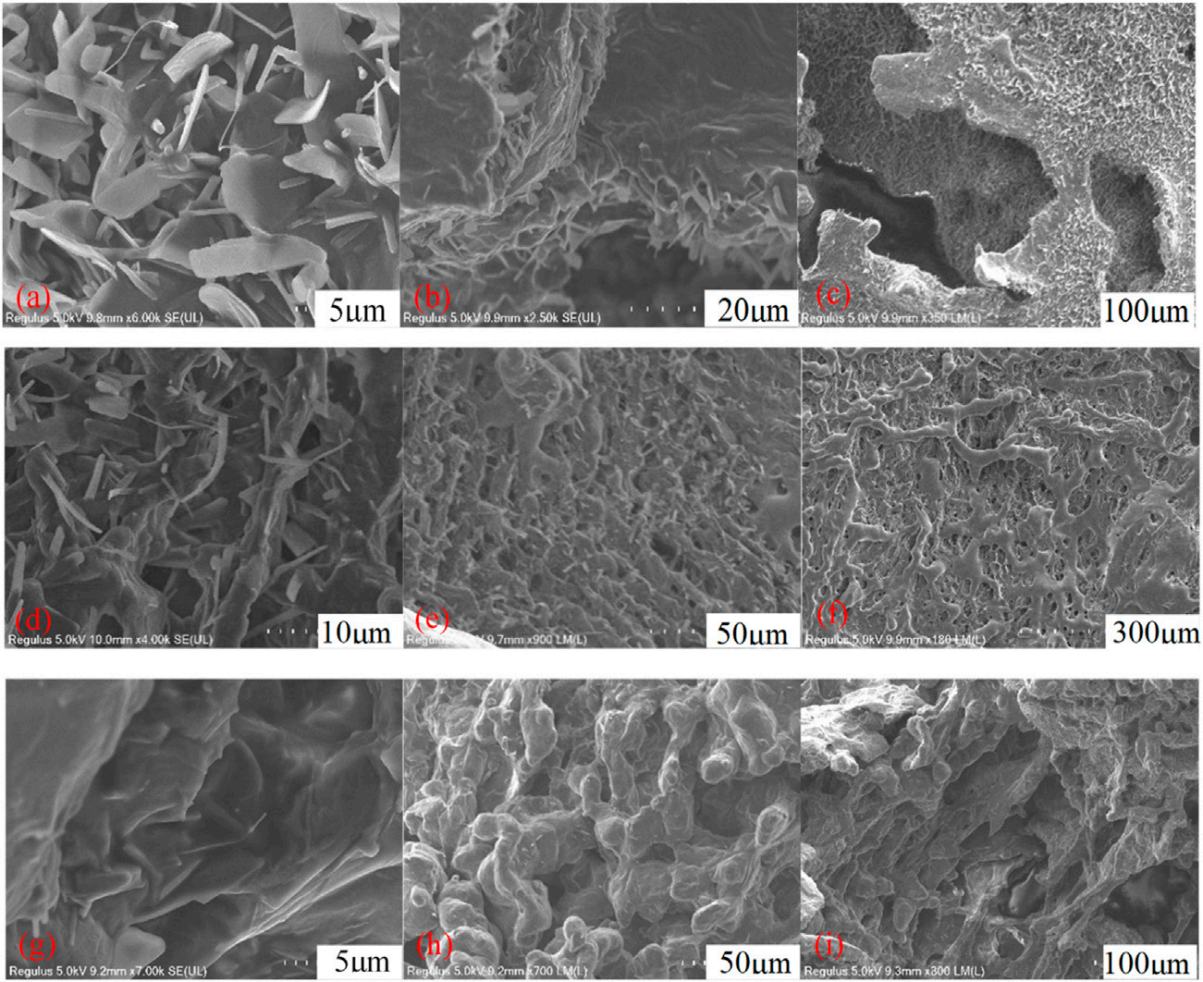
The microstructure of VES-c solution: **(A)** 0.3% VES-c solution aggregated microstructure; **(B)** 0.3% VES-c solution layered microstructure; **(C)** 0.3% VES-c solution overall flake and layered microstructure; **(D)** 1% VES-c solution aggregated microstructure; **(E)** 1% VES-c solution layered microstructure; **(F)** 1% VES-c solution overall microstructure; **(G)** 3% VES-c solution aggregation microstructure; **(H)** 3% VES-c solution worm-like micelle microstructure; **(I)** 3% VES-c solution overall layered microstructure. The effect of VES-c on retarding acid–rock reaction.

To analyze the microstructures of the VES-c surfactant, the 0.3% VES-c, 1% VES-c, and 3% VES-c solutions were observed by the cold-field SEM. Figure 7A shows that the 0.3% VES-c is randomly stacked in the solution as small flakes and slender columns. In the longitudinal direction, the structure is densely stacked and layered (see Figure 7B). The densely layered accumulations connect to the sheets, forming a large gap between the sheets but with no worm-like structure (see Figure 7C). In the 1% VES-c solution, the aggregation state of VES-c changes from chaotic accumulation to a long strip structure formed by small flakes and slender columns (see Figure 7D), and the overlap of long strips forms a layered grid structure (Figure 7E). The overall structures are long strips (some are worm-like shapes) interconnected to form a layered network structure with dense holes (Figure 7F). When the concentration increased to 3%, the aggregation of molecules appears as a large number of small flakes and slender columns formed a folded membrane (Figure 7G). The magnified observation shows a clear worm-like structure (Figure 7H). In the horizontal direction, the structures are entangled and connected, and in the longitudinal direction, the structure is densely layered (Figure 7I).

No worm-like micelles were formed in the 0.3% VES-c solution, but the long-chain tail and Gemini structure of the molecule effectively increased the viscosity of the solution. In the 1% and 3% VES-c solutions, worm-like micelles were formed, and the worm-like micelles were connected horizontally to form a longitudinal layer. This structure is stable and dense. It suggests that VES-c represents a good viscosity increasing effect, temperature resistance, and shear resistance.

### The Effect of VES-c on Retarding Acid–Rock Reaction

To explore the effect of the VES-c retarding acid–rock reaction, we studied the four groups of acid–rock reactions. Table 1 summarized the rock dissolution rate and liquid pH after reaction for four groups. Group 1 is the reaction between dolomite and dibasic acid (3% HCl and 5% CH_3_COOH). Group 2 is the reaction between dolomite and 0.3% VES-c dibasic acid. Group 3 is the reaction of broken core and dibasic acid. Group 4 is the reaction between broken core and 0.3% VES-c dibasic acid. After the dolomite reactions (e.g., Group 1 and Group 2), the pH of solutions is 4.5, and the *k* of Group 2 is 4.45% higher than that of Group-1, indicating that VES-c does not adhere to the surface of the dolomite to hinder the reaction. For the broken core reactions (e.g., Group 3 and Group 4), the pH of solutions is 0.5, and the *k* of Group 4 is 1.29% higher than that of Group 3, indicating that VES-c does not adhere to the surface of the broken core and is beneficial to the acid and broken core reaction.

**TABLE 1:** Results of four acid–rock reaction groups.

Groups	Rocks	*M*_B_ (g)^a^	*M*_A_ (g)^b^	*k* (%)^c^	Acids	pH
1	Dolomite	1.9977	0.9268	53.60	Dibasic acid	4.5
2	Dolomite	2.0020	0.8396	58.06	Dibasic acid + 0.3%VES-c	4.5
3	Broken core	2.0031	1.7464	12.73	Dibasic acid	0.5
4	Broken core	2.0015	1.7208	14.02	Dibasic acid + 0.3%VES-c	0.5

a Rock quality before reaction.

b Rock quality after reaction.

c Rock dissolved ratio k = (M_B_ − M_A_)/M_B_ × 100%; the dibasic acid represents the 3% HCl and 5% CH_3_COOH solutions.

**FIGURE 8:**
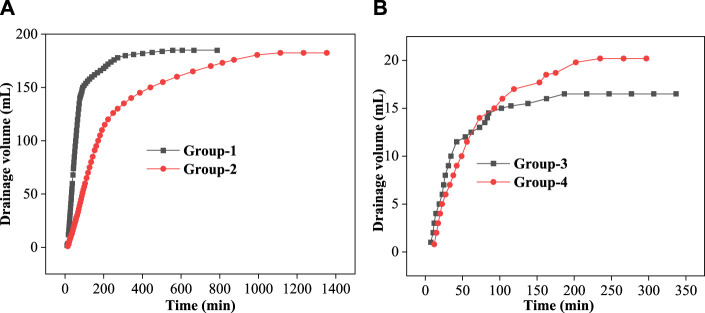
Drainage volume varies with time: **(A)** dolomite reactions; **(B)** core reactions.

Figure 8A shows that the drainage volume varies with time for Group 1 and Group 2. The reaction between dolomite and dibasic acid (black line) was very rapid in the first 90 min, and about 150 ml of water was drained. After 310 min, the reaction ended. However, for the Group 2 reaction (red line), about 50 ml of water was collected in the first 90 min, and the reaction lasted about 1000 min. The reaction rate of Group 2 was retarded, about 66.67%, compared with that of Group-1. It is obvious that the 0.3% VES-c retards the reaction rate of dolomite and dibasic acids. In the same manner, Figure 8B shows that the drainage volume varies with time for Group 3 and Group 4. In the first 90 min, the drainage volume of Group 3 (black line) was slightly greater than that of Group 4 (red line). However, after 90 min, the drainage volume of Group 3 was almost unchanged, but Group 4 reacted for 234 min. The reaction of broken core and dibasic acids was retarded as well.

#### ICP-MS Analysis

**FIGURE 9:**
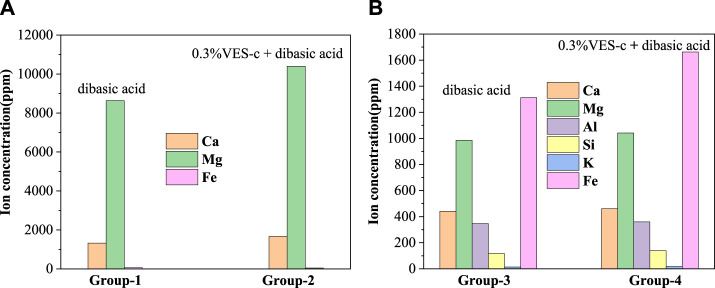
The composition and content of elements in the solution after the acid–rock reaction: **(A)** dolomite reactions; **(B)** core reactions.

To analyze the element contents of solution after acid–rock reaction, an inductively coupled plasma mass spectrometer (ICP-MS) was used to determine the types and contents of elements. Figure 9A shows that the contents of Ca and Mg in the solution of Group 2 are higher than those of Group 1. This is consistent with the result of the dissolved ratio (see Table 1). Notably, the ratio of Mg and Ca (Mg/Ca = 6.2) in Group 2 is lower than that (Mg/Ca = 6.5) of Group 1. In the acid solution of pH ≈ 4.5, the VES-c may decrease the formation of amorphous calcium carbonate (ACC) (Rodriguez-Blanco et al., 2012; Rao et al., 2016), which increases the content of Ca^2+^ in the solution, which is beneficial to reduce reservoir damage. Usually, the chemical composition of dolomite is CaMg(CO_3_)_2_, where Mg is replaced with Fe to produce CaMg_0.77_Fe_0.23_(CO_3_)_2_; thus, there is a small amount of Fe. The reaction equations are as follows:

$$C_{a}\mathrm{Mg(C}O_{3}{)}_{2}\mathrm{+4}H^{+}\;\to \;Ca^{\mathrm{2+}}\mathrm{+M}g^{\mathrm{2+}}\mathrm{+2C}O_{2}↑\mathrm{+2}H_{2}O$$ (1)

$$C_{a}Mg_{0\mathrm{.77}}Fe_{0\mathrm{.23}}{\left(CO_{3}\right)}_{2}\mathrm{+4}H^{+}\;\to \;Ca^{\mathrm{2+}}\mathrm{+0}\mathrm{.77M}g^{\mathrm{2+}}\mathrm{+0}\mathrm{.23F}e^{\mathrm{2+}}\mathrm{+2C}O_{2}↑\mathrm{+2}H_{2}O$$ (2)

In Figure 9B, the main elements are Fe, Mg, Ca, and Al. The carbonate minerals in the core are iron calcite and iron dolomite, so the content of Fe is the highest. The content of each element of Group 4 is higher than that of Group 3, which is consistent with the dissolved ratio. The reaction equations between the main minerals contained in the core and the acid solution are as follows.

The related reactions of feldspar minerals and acid solution are as follows:

$$\left(\mathrm{Na, K}\right)\mathrm{AlS}i_{3}O_{8}\mathrm{+4}H^{+}\mathrm{+4}H_{2}O\;\to \;3H_{4}\mathrm{Si}O_{4}\mathrm{+(Na, K}{)}^{+}\mathrm{+A}l^{\mathrm{3+}}$$ (3)

$$CaAl_{2}Si_{2}O_{8}\mathrm{+8}H^{+}\;\to \;2H_{4}\mathrm{Si}O_{4}\mathrm{+C}a^{\mathrm{2 +}}\mathrm{+2A}l^{\mathrm{3+}}$$ (4)

The reactions of carbonate minerals and acid solution are shown in Eqs. 2, 5:

$$\mathrm{CaFe}{\left(CO_{3}\right)}_{2}\mathrm{+4}H^{+}\;\to \;Ca^{\mathrm{2+}}\mathrm{+F}e^{\mathrm{2+}}\mathrm{+2C}O_{2}↑\mathrm{+2}H_{2}O$$ (5)

The clay mineral illite is relatively stable and hardly reacts with acid at room temperature. The chemical components of chlorite are (Mg,Fe,Al)_3_ [(Si,Al)_4_O_10_](OH)_8_ and (Mg, Fe, Al)_3_(OH)_6_, and the main chemical components are SiO_2_, A1_2_O_3_, FeO, and MgO. The corresponding reactions are shown in the following equations:

$$A1_{2}O_{3}\mathrm{+6}H^{+}\to \mathrm{2A}l^{\mathrm{3+}}\mathrm{+3}H_{2}O$$ (6)

$$\mathrm{FeO+2}H^{+}\;\to \;Fe^{\mathrm{2+}}+H_{2}O$$ (7)

$$\mathrm{MgO+2}H^{+}\;\to \;Mg^{\mathrm{2+}}+H_{2}O$$ (8)

#### SEM-EDS Analysis

**FIGURE 10:**
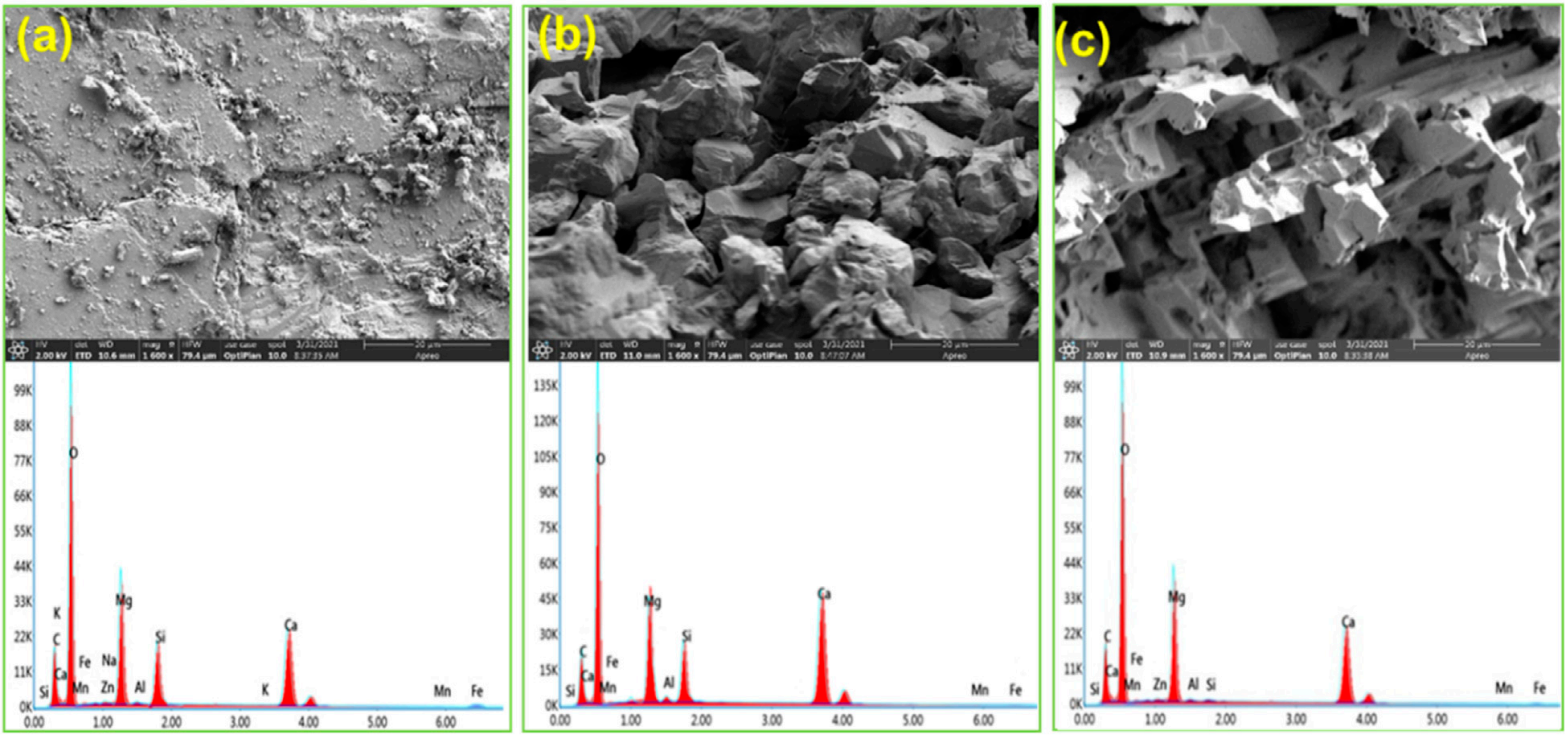
SEM-EDS analysis of acid-dolomite etching: **(A)** untreated dolomite; **(B)** dolomite etched by dibasic acid; **(C)** dolomite etched by 0.3% VES-c dibasic acid. The top is the SEM figure and the corresponding bottom is the EDS figure.

Figures 10A–C show the results of the scanning electron microscope (SEM) and energy dispersive spectrometer (EDS) for the dolomite surface. There are not obvious pores and cracks on the surface of the untreated dolomite. However, after the reaction, pores and cracks are observed on the dolomite surfaces. More importantly, the dolomite acidified by 0.3% VES-c dibasic acid has more and even pores and cracks on the surface (*see* Figures 10B,C). In addition, comparing with the EDS of Figures 10A–C, the difference of elements is small, which proves that VES-c is not easy to adhere to the dolomite surface. Otherwise, the C element would increase greatly.

**FIGURE 11:**
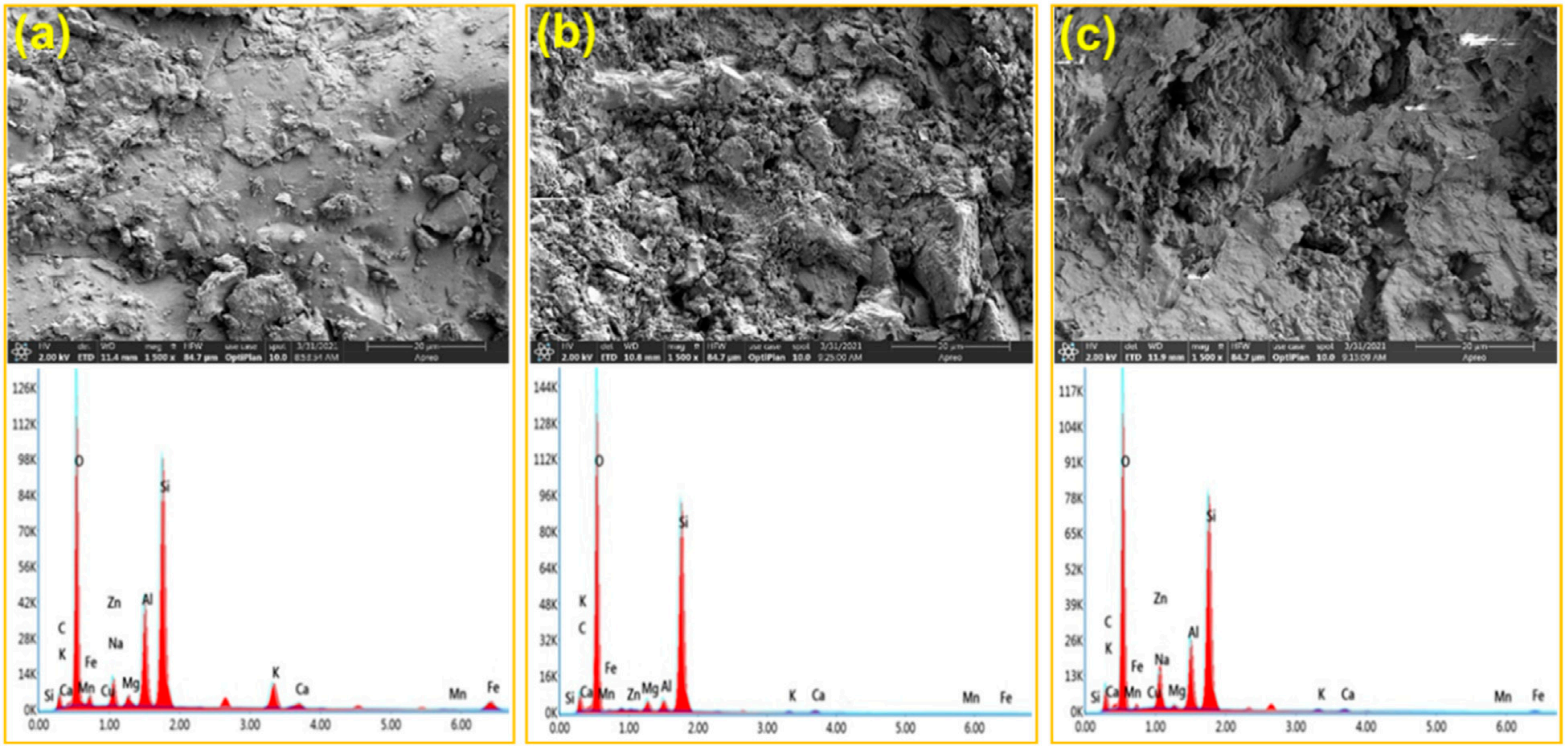
SEM-EDS analysis of acid-core etching: **(A)** untreated core; **(B)** core etched by dibasic acid; **(C)** core etched by 0.3% VES-c dibasic acid. The top is the SEM figure and the corresponding bottom is the EDS figure.

The SEM-EDS result of acid-core etching is shown in Figures 11A–C. Comparing Figure 11B with Figure 11C, the core surface after the dibasic acid treatment looks messy, loose, and fragile. Moreover, the core surface after 0.3% VES-c dibasic acid treatment is relatively regular and firm. It suggests that VES-c is favorable to deep acidification of rock and prevents loose particles from clogging pores. In addition, by comparing the EDS figures, the Ca, Fe, and Mg of the reacted cores are reduced and the difference in the C element is small, which proves that the carbonate minerals reacted and the VES-c is not easy to adhere to the core surface.

#### XRD Analysis

**FIGURE 12:**
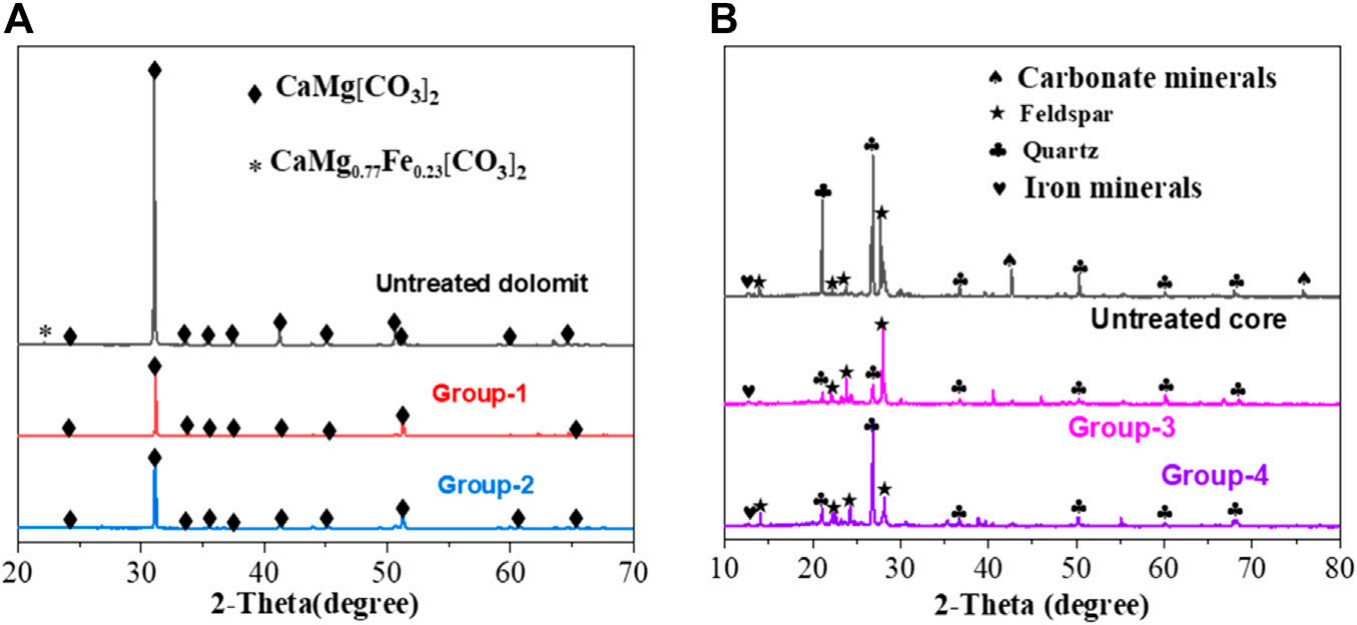
Solid XRD analysis after dissolution: **(A)** comparison of untreated dolomite and Group 1 and Group 2 reactions; **(B)** comparison of untreated cores and Group 3 and Group 4 reactions.

The effect of VES-c was analyzed from a microscopic view by the SEM-EDS. To fully investigate the effect of VES-c, the XRD analysis was also performed from a macroscopic view. The results show that the peak intensity of dolomite is reduced after the reaction for Group 1 and Group 2, and some peaks disappear. This is because the contents of Ca, Mg, and Fe in the dolomite are changed (Figure 12A). For Group 3 and Group 4, the carbonate mineral peaks of the cores disappeared after the reaction, indicating that the reaction finished. Adding 0.3% VES-c would not affect the dissolution of dibasic acid on the dolomite and core (*see* Figure 12B).

### The Mechanism of VES-C Retarding Acid–Rock Reaction

0.3% VES-c retards the acid–rock reaction because the solution viscosity is increased without adhering to the core surface. There are three effects of 0.3% VES-c acid viscous solution. First, the movement of H^+^ in the solution is slowed. Second, the viscous liquid reduces the fluid loss and increases the spreading area of the liquid (Yu et al., 2019b), thereby resulting in a uniform and deep acidification. Third, the viscous liquid restrains the overflow of CO_2_ (see Supplementary Figure S4). The CO_2_ in the solution extends the distance of H^+^ to the solid surface and is tethered at the solid surface to reduce the touch efficiency of H^+^. In addition, the amount of CO_2_ increases in the solution; namely, the increase of product concentration reduces the reaction rate.

The VES-c with good salt tolerance is not precipitated with ion concentration increasing during the reaction. The total concentration of Ca^2+^, Mg^2+^, Fe^2+^, and Al^3+^ in the solution after Group 4 reaction reached 3085 mg/L, indicating that VES-c has good resistance to high-valent ions.

## Conclusion

In this work, we synthesized a Gemini zwitterionic viscoelastic surfactant (VES-c) with good acid and salt resistance, temperature resistance, and shear resistance. Although no worm-like micelle structure was formed in the 0.3% VES-c solution, the viscosity of 0.3%VES-c dibasic acid (3% HCl+5% CH_3_COOH) increases due to its special molecular structure forming the layer structure with pores. A self-assembled device was used to verify the effect of 0.3% VES-c on retarding the reaction of dibasic acid and rock. ICP-MS, SEM-EDS, and XRD were used to verify element content and structure after the acid–rock reaction. The main conclusions are obtained as follows:

1) 0.3% VES-c can prolong the reaction time and ICP-MS results show that the ion concentrations in the solution for Group 1/ 2 or Group 3/ 4 reactions are similar, which suggests that VES-c is not easy to adhere to the solid surface. In addition, VES-c decreases the formation of ACC.
2) SEM-EDS intuitively exhibits that 0.3% VES-c dibasic acid better dissolves dolomite and cores, and the dissolved solids are more uniform and produce more pores with harmless solids. XRD also verifies the effect of 0.3% VES-c in enhancing acid–rock dissolution.
3) The mechanism of VES-c retarding the acid–rock reaction was analyzed. First, 0.3% VES-c increases the viscosity of the dibasic acid and does not adhere to the solid surface. Second, the viscous VES-c solution inhibits the H^+^ movement, reducing the solution and filtering out and expanding the spreading area of the liquid. Third, the viscous VES-c solution also restrains CO_2_ to escape from the liquid, thereby extending the distance of H^+^ movement, reducing the touch area of solids, and thus reducing the reaction rate.

## Data Availability

The original contributions presented in the study are included in the article/Supplementary Material; further inquiries can be directed to the corresponding authors.

## References

[B1] Afra S. Samouei H. Truong P. Nasr-El-Din H. (2020). Micellar Growth and Network Formation in Acidic Solutions of a Sulfobetaine Zwitterionic Surfactant Triggered by an Inorganic Salt. Soft Matter 16, 4494–4501. 10.1039/d0sm00399a 32338671

[B2] Asadollahpour E. Hashemolhosseini H. Baghbanan A. Mohtarami E. (2019). Redistribution of Local Fracture Aperture and Flow Patterns by Acidizing. Int. J. Rock Mech. Mining Sci. 117, 20–30. 10.1016/j.ijrmms.2019.03.018

[B3] Assem A. I. Kumar H. T. Nasr-El-Din H. A. De Wolf C. A. (2019). Location and Magnitude of Formation Damage Due to Iron Precipitation during Acidizing Carbonate Rocks. J. Pet. Sci. Eng. 179, 337–354. 10.1016/j.petrol.2019.04.073

[B4] Buijse M. A. van Domelen M. S. (2000). Novel Application of Emulsified Acids to Matrix Stimulation of Heterogeneous Formations. SPE 15, 208–213. 10.2118/65355-pa

[B5] Bulgakova G. T. Kharisov R. Y. Pestrikov A. V. Sharifullin A. R. (2013). Experimental Study of a Viscoelastic Surfactant-Based In Situ Self-Diverting Acid System: Results and Interpretation. Energy Fuels 28, 1674–1685. 10.1021/ef4019542

[B6] Cai C. B. Xu Y. Wang X. He C. Gao Y. Duan G. (2018). A New High Temperature Polymer Fracturing Fluid. IOP Conf. Ser. Earth Environ. Sci. 186. 10.1088/1755-1315/186/4/012028

[B7] Chen J. Song B. Pei X. Cui Z. Xie D. (2019). Rheological Behavior of Environmentally Friendly Viscoelastic Solutions Formed by a Rosin-Based Anionic Surfactant. J. Agric. Food Chem. 67, 2004–2011. 10.1021/acs.jafc.8b06985 30715867

[B8] Dehdari B. Parsaei R. Riazi M. Rezaei N. Zendehboudi S. (2020). New Insight into Foam Stability Enhancement Mechanism, Using Polyvinyl Alcohol (PVA) and Nanoparticles. J. Mol. Liquids 307, 112755. 10.1016/j.molliq.2020.112755

[B9] Guo T. Li Y. Ding Y. Qu Z. Gai N. Rui Z. (2017). Evaluation of Acid Fracturing Treatments in Shale Formation. Energy Fuels 31, 10479–10489. 10.1021/acs.energyfuels.7b01398

[B10] Israelachvili J. N. D. J. Mitchell B. W. (1976). Theory of Self-Assembly of Lipid Bilayers and Vesicles. Biochim. Biophys. Acta. 470, 185–201. 10.1016/0005-2736(77)90099-2 911827

[B11] Jeffry S. J. M. Trjangganung K. Chandrakant A. A. Madon B. Katende A. Ismail I. (2020). Selection of Suitable Acid Chemicals for Matrix Stimulation: A Malaysian Brown Field Scenario. J. Pet. Sci. Eng. 186, 1–21. 10.1016/j.petrol.2019.106689

[B12] Jones A. T. Dovle M. (1996). Improving the Efficiency of Matrix Acidizing with a Succinoglycan Viscosifier. SPE 11, 144–149. 10.2118/30122-pa

[B13] Liu Y. Li H. (2016). Application of a Novel Hyperbranched-Polymer Fracturing-Fluid System in a Low-Permeability Heavy-Oil Reservoir. SPE 174461, 1–12. 10.2118/174461-pa

[B14] Lu Y. Yang M. Ge Z. Zhou Z. Chai C. Zhao H. (2019). Influence of Viscoelastic Surfactant Fracturing Fluid on Coal Pore Structure Under Different Geothermal Gradients. J. Taiwan Inst. Chem. Eng. 97, 207–215. 10.1016/j.jtice.2019.01.024

[B15] Marrugo-Hernandez J. J. Prinsloo R. Sunba S. Marriott R. A. (2018). Downhole Kinetics of Reactions Involving Alcohol-Based Hydraulic Fracturing Additives with Implications in Delayed H2s Production. Energy Fuels 32, 4724–4731. 10.1021/acs.energyfuels.7b04036

[B16] Mejia L. Tagavifar M. Xu K. Mejia M. Du Y. Balhoff M. (2019). Surfactant Flooding in Oil-Wet Micromodels with High Permeability Fractures. Fuel 241, 1117–1128. 10.1016/j.fuel.2018.12.076

[B17] Pak. A. Chan D. H. (2004). A Fully Implicit Single-phase T-H-M Fracture Model for Modelling Hydraulic Fracturing in Oil Sands. J. Can. Petrol. Tech. 43, 35–44. 10.2118/04-06-01

[B18] Qu M. Hou J. Liang T. Raj I. Yang Y. Qi P. (2020). Synthesis of α-starch Based Nanogel Particles and its Application for Long-Term Stabilizing Foam in High-Salinity, High-Temperature and Crude Oil Environment. J. Petrol. Sci. Eng. 191, 107185. 10.1016/j.petrol.2020.107185

[B19] Rao A. Vásquez-Quitral P. Fernández M. S. Berg J. K. Sánchez M. Drechsler M. (2016). pH-Dependent Schemes of Calcium Carbonate Formation in the Presence of Alginates. Cryst. Growth Des. 16, 1349–1359. 10.1021/acs.cgd.5b01488

[B20] Rbeawi S. A. Kadhim F. S. Farman G. M. (2018). Optimum Matrix Acidizing: How Much Does it Impact the Productivity. IOP Conf. Ser. Mater. Sci. Eng. 454, 012105. 10.1088/1757-899x/454/1/012105

[B21] Rodriguez-Blanco J. D. Shaw S. Bots P. Roncal-Herrero T. Benning L. G. (2012). The Role of pH and Mg on the Stability and Crystallization of Amorphous Calcium Carbonate. J. Alloys Comp. 536, S477–S479. 10.1016/j.jallcom.2011.11.057

[B22] Sayed M. A. Al-Muntasheri G. A. (2016). Mitigation of the Effects of Condensate Banking: A Critical Review. SPE 31, 85–102. 10.2118/168153-pa

[B23] Sayed M. A. Nasr-Ei-Din H. A. Zhou J. Zhang L. Holt S. (2012). A New Emulsified Acid to Stimulate Deep Wells in Carbonate Reservoirs: Coreflood and Acid Reaction Studies. SPE 151062, 1–30. 10.2118/151062-ms

[B24] Tangirala S. Sheng J. J. (2019). Roles of Surfactants During Soaking and Post Leak-Off Production Stages of Hydraulic Fracturing Operation in Tight Oil-Wet Rocks. Energy Fuels 33, 8363–8373. 10.1021/acs.energyfuels.9b01913

[B25] Wang J. Huang Y. Zhou F. Song Z. Liang X. (2020). Study on Reservoir Damage During Acidizing for High-Temperature and Ultra-deep Tight Sandstone. J. Petrol. Sci. Eng. 191, 107231. 10.1016/j.petrol.2020.107231

[B26] Wang X. C. Jia Y. Z. Wang B. Wang P. Xu Z. D. Wu G. F. (2016). Research and Application on Low Molecular Weight Polymer Fracturing Fluid System. MATEC Web of Conferences 63, 1–4. 10.1051/matecconf/20166303019

[B27] Yang J. Hou J. (2020). Synthesis of Erucic Amide Propyl Betaine Compound Fracturing Fluid System. Coll. Surf. A: Physicochem. Eng. Aspects 602, 125098. 10.1016/j.colsurfa.2020.125098

[B28] Yang Y. H. Hu D. Huang Y. Z. (2006). A New Stimulating Technique of Sandstone Reservoir: Acid Fracturing. Fault Block Oil & Gas Field 13, 78–80. 10.3969/j.issn.1005-8907.2006.03.026

[B29] Yu Q. Liang S. Tan S. Sun Z. Yu Y. (2019). Experimental Study on Surface-Active Polymer Flooding for Enhanced Oil Recovery: A Case Study of Daqing Placanticline Oilfield, NE China. Petrol. Explor. Develop. 46, 1138–1147. 10.1016/s1876-3804(19)60274-0

[B30] Yu X. Li Y. Liu Y. Yang Y. Wu Y. (2019). Flow Patterns of Viscoelastic Fracture Fluids in Porous Media: Influence of Pore-Throat Structures. Polymers 11, 1291. 10.3390/polym11081291 PMC6723620 31382385

[B31] Zakaria A. S. Nasr-Ei-Din H. A. (2015). A Novel Polymer Assisted Emulsified Acid System Improves the Efficiency of Carbonate Acidizing. SPE 173711, 1–24. 10.2118/173711-ms

[B32] Zhang S. Fang Z. (2020). Permeability Damage Micro-mechanisms and Stimulation of Low-Permeability Sandstone Reservoirs: A Case Study from Jiyang Depression, Bohai Bay Basin, China. Pet. Exploration Dev. 47, 374–382. 10.1016/s1876-3804(20)60054-4

[B33] Zhang W. Mao J. Yang X. Zhang H. Zhang Z. Yang B. (2018). Study of a Novel Gemini Viscoelastic Surfactant with High Performance in Clean Fracturing Fluid Application. Polymers 10, 1215. 10.3390/polym10111215 PMC6290609 30961140

[B34] Zhang W. Mao J. Yang X. Zhang H. Zhao J. Tian J. (2019). Development of a Sulfonic Gemini Zwitterionic Viscoelastic Surfactant with High Salt Tolerance for Seawater-Based Clean Fracturing Fluid. Chem. Eng. Sci. 207, 688–701. 10.1016/j.ces.2019.06.061

[B35] Zhang Y. Mao J. Zhao J. Yang X. Zhang Z. Yang B. (2018). Preparation of a Novel Ultra-high Temperature Low-Damage Fracturing Fluid System Using Dynamic Crosslinking Strategy. Chem. Eng. J. 354, 913–921. 10.1016/j.cej.2018.08.021

[B36] Zhang Z. Mao J. Yang X. Zhao J. Smith G. S. (2018). Advances in Waterless Fracturing Technologies for Unconventional Reservoirs. Energy Sourc. A: Recovery, Utilization, Environ. Effects 41, 237–251. 10.1080/15567036.2018.1514430

[B37] Zhou F. Su H. Liang X. Meng L. Yuan L. Li X. (2019). Integrated Hydraulic Fracturing Techniques to Enhance Oil Recovery from Tight Rocks. Pet. Exploration Dev. 46, 1065–1072. 10.1016/s1876-3804(19)60263-6

