# Supplementary material for: Synthesis and Characterization of Surfactant for Retarding Acid–Rock Reaction Rate in Acid Fracturing
Source: Front Chem. 2021 Aug 20;9:715009. doi: 10.3389/fchem.2021.715009 (PMC8417810; doi:10.3389/fchem.2021.715009)
Supplement: Supplementary file 1 [file DataSheet1.docx]

Supplementary Material

**Table S1**. Chemical composition of each mineral

| Name | Chemical composition |
| --- | --- |
| Illite | {K_2_[Al(Fe^3+^),Mg][(Si·Al)_4_O_10_]} *n*H_2_O |
| Chlorite | (Mg,Fe,Al)_3_[(Si,Al)_4_O_10_](OH)_8_+(Mg、Fe、Al)_3_(OH)_6_ |
| Montmorillonite | {E_0.33_}(A1_5/3_Mg_1/3_)[Si_4_O_10_](OH)_2_·*n*H_2_O |
| Kaolinite | Al_4_[Si_4_O_10_](OH)_8_ |
| Calcite | CaCO_3_ |
| laumontite | Ca[AlSi_2_O_6_]_2_﹒4H_2_O |
| Dolomite | (Ca,Mg)(CO_3_)_2_ |
| Quartz | SiO_2_ |
| Feldspar | *x*[AlSi_3_O_8_],*x*=Na、K;CaAl_2_Si_2_O_8_ |
| Muscovite | K{Al_2_[AlSi_3_O_10_][OH]_2_} |

**Materials and methods**

**Materials**

Erucamidopropyl dimethylamine was bought from Shanghai Winson New Material Technology Co., Ltd. (Shanghai, China); glycine, ethanol, epichlorohydrin, sodium hydroxide, hydrochloric acid, acetic acid, sodium chloride, 8-10 million molecular weight polyacrylamide were purchased from Shanghai Macklin Biochemical Technology Co., Ltd. (Shanghai, China). Dolomite, the particle size of 0.5-1 cm, was purchased from Dongguan Ruiheng Mineral Products Co., Ltd. (Guangzhou, China). The core fragments sample were taken from the Yuan 284 block of Changqing Oilfield, China. The detrital components were mainly feldspar (average, 34.82%) and quartz (average, 29.33%). Carbonates were mainly iron calcite (average, 2.7%) and iron dolomite (1.5%); Clay minerals were mainly illite (average 6.7%) and chlorite (2.4%); siliceous materials are mainly quartz (1.02%), occasionally pyrite (0.02%) and ridge iron are (0.04%).

**Methods**

**FT-IR**

FT-IR analysis of the VES-c was performed using the VECTOR-22 FT-IR spectrometer (Bruker, Germany). The sample was prepared in potassium bromide. Analysis of the sample was performed scanning through 400-4000 cm^-1^ wavenumber range.

**NMR**

NMR analysis of the VES-c was performed using the Avance Neo 600MHz spectra (Bruker, Germany).


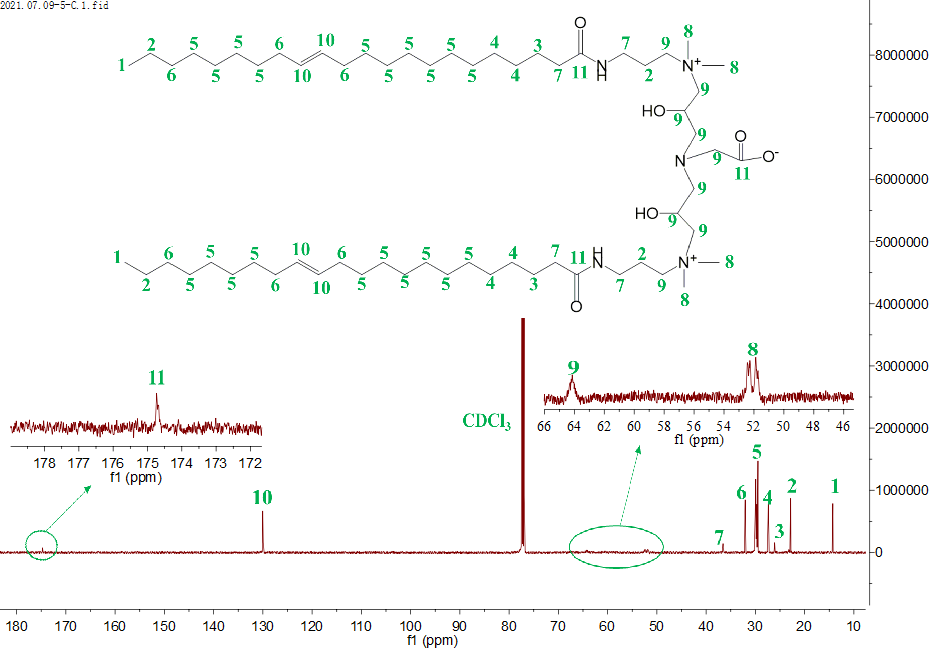


**Figure S1**. ^13^C NMR of VES-c

**Surface tension**

The SFZL-A1 surface tension tester (Shanghai, China) was used to measure the surface tension of the VES-c at 25 °C. The measurement method was the platinum plate method, and the accuracy and repeatability error was 0.1 mN/m.

**Dissolution**

At room temperature, observe the dissolution of the 0.3% VES-c, 1.5% VES-c, 2.7% VES-c in deionized water, 2% HCl, 3% HCl, 5% HCl, 6% HCl, 8% HCl, 9% HCl, 0.5% NaCl, 1% NaCl, 1.5% NaCl, 2% NaCl solution to verify its acid and salt resistance.

**Viscosity**

At room temperature, the NDJ-8S digital rotational viscometer (Jiangsu, China) was used to measure the viscosity of the VES-c solution with 0.1% increments and the concentration from 0.1% to 1%. And we compare the viscosity of polyacrylamide with a molecular weight of 8-10 million under the same concentration. The rotor was NO.2 and the RPM was 12.

**Shear-resistance**

The dynamic rheometer (DHR-1, TA Company, USA) was used to determine the shear resistance of VES-c at170s^-1^of shear rate and 95°C. The temperature was raised from 25°C to 95°C, and keep a constant shearing at 95°C for 1h.

**Temperature-resistance**

The temperature resistance performance of VES-c was measured using the synchronous comprehensive thermal analyzer (Model STA409PC, Netzsch, Germany). The test condition was starting from 40 ℃, heating up to 400 ℃ at a heating rate of 10 ℃/min. The decomposition temperature of VES-c and the change of VES-c mass with temperature were determined according to the TG-T-DTA curve, and the VES-c temperature resistance was found as well.

**Microstructure**

Using SU8100 cold field emission scanning electron microscope (HITACHI, Japan) to observe the microstructure of 0.3%VES-c, 1% VES-c, and 3% VES-c. The samples were prepared by the freeze-drying method. The samples were quickly frozen with liquid nitrogen, and then the frozen samples were placed under high vacuum conditions to sublimate the moisture in the sample. The moisture was directly converted from solid to gas to avoid or reduce damaging the structure caused by the freeze-drying process, and ensuring the microstructure of VES-c in the solution can be observed. After the freeze-drying process, the samples were conductively coated by spraying Au.

**Effect of VES-c on retarding acid-rock reaction**

After the acid-rock reaction was completed, the elemental composition and content in the solution after the reaction determined by the inductively coupled plasma mass spectrometer (ICP-MS) (Agilent, USA). To analyze and verify the effect of VES-c on retarding acid-rock reaction, the scanning electron microscope (SEM-EDS) (FEI, USA) was used to observe the surface of dolomite and core before and after acid dissolution. The EDS can verify whether VES-c would be adsorbed on the surface of dolomite or core. Finally, the in-situ X-ray diffractometer (Bruck, Germany) was used to analyze the dolomite and core before and after the acid dissolution, and it is further proved that VES-c contributes to the acid-rock reaction from the macro perspective.

**VES-c dissolution**

Different concentrations of VES-c are well dissolved in deionized water as shown in Figure S2(a). Figures S2(b) – 5(d) show that the VES-c can be dissolved in the 2% HCl, 3% HCl and 5% HCl and significantly increase the viscosity of the acid. As the VES-c concentration increases, the acid solution becomes viscous.

Figure S2(e) – (h) show that the VES-c represents good solubility in the 0.5% – 2% NaCl solutions. The concentration of surfactant is greater, and the effect of thickening is more obvious, indicating that VES-c has good salt tolerance.


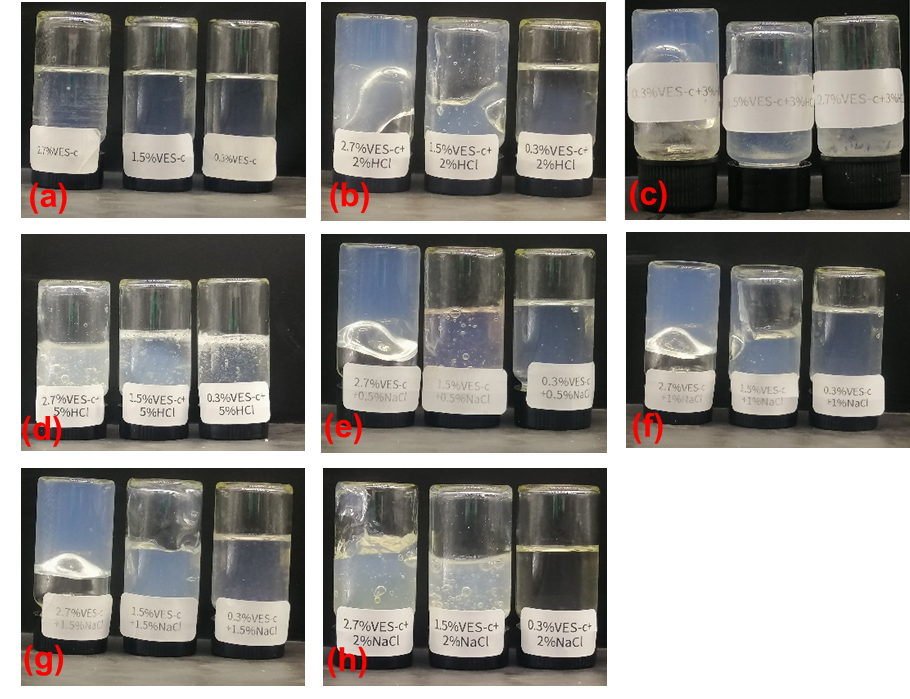


**Figure S2**. Dissolution of 0.3% VES-c, 1.5% VES-c, and 2.7% VES-c in different solution: (a) deionized; (b) 2% HCl solution; (c) 3% HCl solution; (d) 5% HCl solution;(e) 0.5% NaCl solution; (f) 1% NaCl solution; (g) 1.5% NaCl solution; (h) 2% NaCl solution.


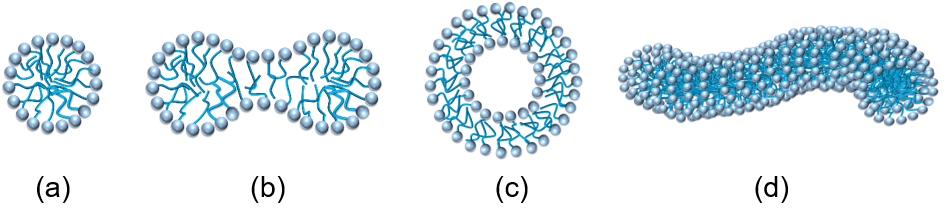


**Figure S3**. Aggregation state of surfactant molecules in dilute solution: (a) spherical micelle; (b) rod-shaped micelles; (c) spherical bilayer vesicle; (d) worm-like micelles.


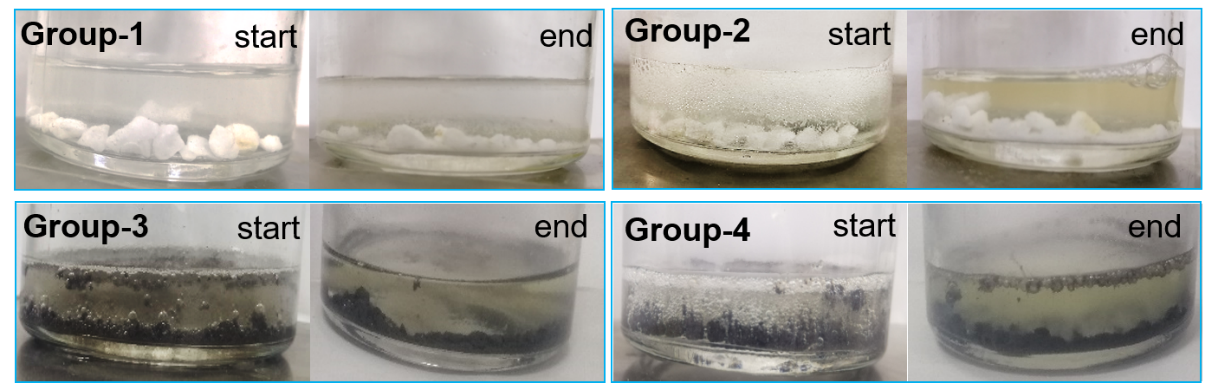


**Figure S4**. The phenomenon of acid-rock reaction.

The 0.3% VES-c dibasic acid solutions have a lot of bubbles during the reaction (see Figure S4 of Group-2 and Group-4), while the dibasic acid solutions without VES-c have no bubbles or few bubbles during the reaction (see Figure S4 of Group-1 and Group-3). After the reaction, there are no bubbles in the solution. The CO_2_ in the solution extends the distance of H^+^ to the solid surface and is tethered at the solid surface to reduce the touch efficiency of H^+^, which is favorable to reduce the acid-rock reaction rate.
